# Revised version of the Cenozoic Collision along the Zagros Orogen, Insights from Cr-spinel and Sandstone Modal Analyses

**DOI:** 10.1038/s41598-017-11042-1

**Published:** 2017-09-07

**Authors:** Parisa Gholami Zadeh, Mohammad Hossein Adabi, Ken-ichiro Hisada, Mahboubeh Hosseini-Barzi, Abbas Sadeghi, Mohammad Reza Ghassemi

**Affiliations:** 1 Department of Geology, Faculty of Earth Sciences, Shahid Beheshti University, Tehran, Iran; 2 Graduate School of Life and Environmental Sciences, University of Tsukuba, Tsukuba, Japan; 3 Research Institute for Earth Sciences, Geological Survey of Iran, Tehran, Iran

## Abstract

Geoscientists have always considered the Neyriz region, located along the Zagros Suture Zone, an important area of interest because of the outcrops of Neotethys ophiolitic rocks. We carried out a modal analysis of the Cenozoic sandstones and geochemistry of the detrital Cr-spinels at Neyriz region in order to determine their provenance and tectonic evolution in the proximal part of Zagros Basin. Our data shows a clear change in provenance from the Late Cretaceous onwards. As from the Late Cretaceous to Eocene, lithic grains are mostly chert and serpentinite; and higher Cr# values of the detrital Cr-spinel compositions indicate that they originate from the fore-arc peridotites and deposited in an accretionary prism setting during this period. From the Late Oligocene to the Miocene periods, volcaniclastic and carbonate lithic grains show an increasing trend, and in the Miocene, metasedimentary lithic grains appear in the sediments. Ophiolite obduction caused a narrow trough sub-basin to be formed parallel to the general trend of the Zagros Orogeny between the Arabian and Iranian plates in Oligocene. From the Miocene onwards, the axial metamorphic complex belt was uplifted in the upper plate. Therefore, the collision along the Zagros Suture Zone must have occurred in the Late Oligocene.

## Introduction

A foreland basin is a basin parallel to the orogenic belt on the continental crust between adjacent craton and the suture zone^1^ which is formed by flexural subsidence caused by topographic load of thrust belt and sedimentary load. Based on the subduction polarities, orogen type (oceanic versus continental crust) and geometries of plate convergence, eight scenarios are considered for sedimentary fills in different setting including: Indo-Burman-type subduction complexes, Apennine-type thin-skinned orogens, Oman-type obduction orogens, Andean-type cordilleras, and Alpine-type collision orogens^2, 3^ in which different rock types are exposed and different sandstone compositions are formed.

The sandstone composition depends on tectonic setting and was known as provenance (e.g. refs 2–4). Dickinson^4^ used three major components of sandstones - quartz, feldspar, and lithic grains - to define tectonic setting by studying recent sandstone compositions. Dickinson’s triangular diagram indicates provenance, which are generally include convergent or divergent setting. As the orogeny field in the Dickinson’s diagram involves all type of collisions, the dynamic interpretation of the peculiar detrital modes and heavy mineral studies is used for a better understanding of the complexity and the type of convergence^2, 3^. Generally, the sediments in an orogenic setting can be derived from five original provenances including; magmatic arc, ophiolite, axial belt metamorphic rocks, continental block, and recycled clastics. Mixed detrital signatures are produced by the erosion of different rock types through geologic periods and space in the orogenic belts. A detailed study of them can reveal when and how the collision occurred^2, 3^.

Detrital Cr-spinel is one of the major opaque heavy minerals in the sedimentary rocks, which is derived from the ultramafic and mafic rocks. It has a good chemical stability and mechanical durability during weathering, transportation and diagenesis; thus, it can be used as an index mineral for studying mantle peridotites^5–11^. The Cr-spinel compositions indicate the degree of partial melting of mantle peridotites^12^. Detrital Cr-spinels are very common in outcrop areas of ophiolites. Therefore, the study of detrital Cr-spinels in the suture zones and fore-arc basins is a useful guide for determining tectonic setting of the sediment provenance^6, 13^. For example, Najman and Garzanti^13^ trace the initial stage of Himalayan collision in northern India based on the sandstone studies and detrital Cr-spinel geochemistry. Cookenboo *et al*.^6^ have used the detrital Cr-spinel geochemistry to reconstruct the tectonic setting for orogeny in the Canadian Cordillera. Detrital Cr-spinel geochemistry can provide useful information about the ultramafic and mafic rocks, which were outcropped, completely eroded and transported to the adjacent basins^9^..

The Zagros Foreland Basin has been formed by the collision of Arabian and Iranian plates during Cenozoic Era due to eastward subduction of Arabian oceanic lithosphere beneath Iranian continental lithosphere. However there are some different ideas about the collision time based on different evidence such as the sequences of deformation, the regional uplift, stratigraphic framework, the chronology of cooling events, topographic evolution and exhumation history (Cretaceous: refs 14–18; Miocene: refs 19 and 20; the Late Eocene to the Oligocene: refs 21–27). Ophiolite obduction occurred in the Late Cretaceous^18, 28^. However, some researchers suggest that the ophiolite emplacement was a diachronous event, and ranged from the Late Cretaceous to the Eocene^29, 30^. It seems that different ideas about the Zagros collision are due to different continental rheology, plate kinematic, rift inheritance and driving forces along the Zagros collisional plate boundary^26^.

The post-collisional sediments record the evolution of the Zagros Orogeny through time. In suture belts, type and geological history of the convergent plates control the provenance^4, 13^. The aim of this study is to determine the provenance and tectonic evolution of the proximal sediments of Zagros Orogeny from the earliest period after Neotethys ophiolite obduction (the Late Cretaceous to the Miocene) using modal analysis of sandstones and Cr-spinel geochemistry in Neyriz region. These kinds of study utilizing the tracking of rock outcrops through time can unravel paleotectonic evaluation of the Zagros Orogeny, and are useful in determining the inception of the Arabia Eurasia collision.

## Geological and tectonic setting

**Figure 1:**
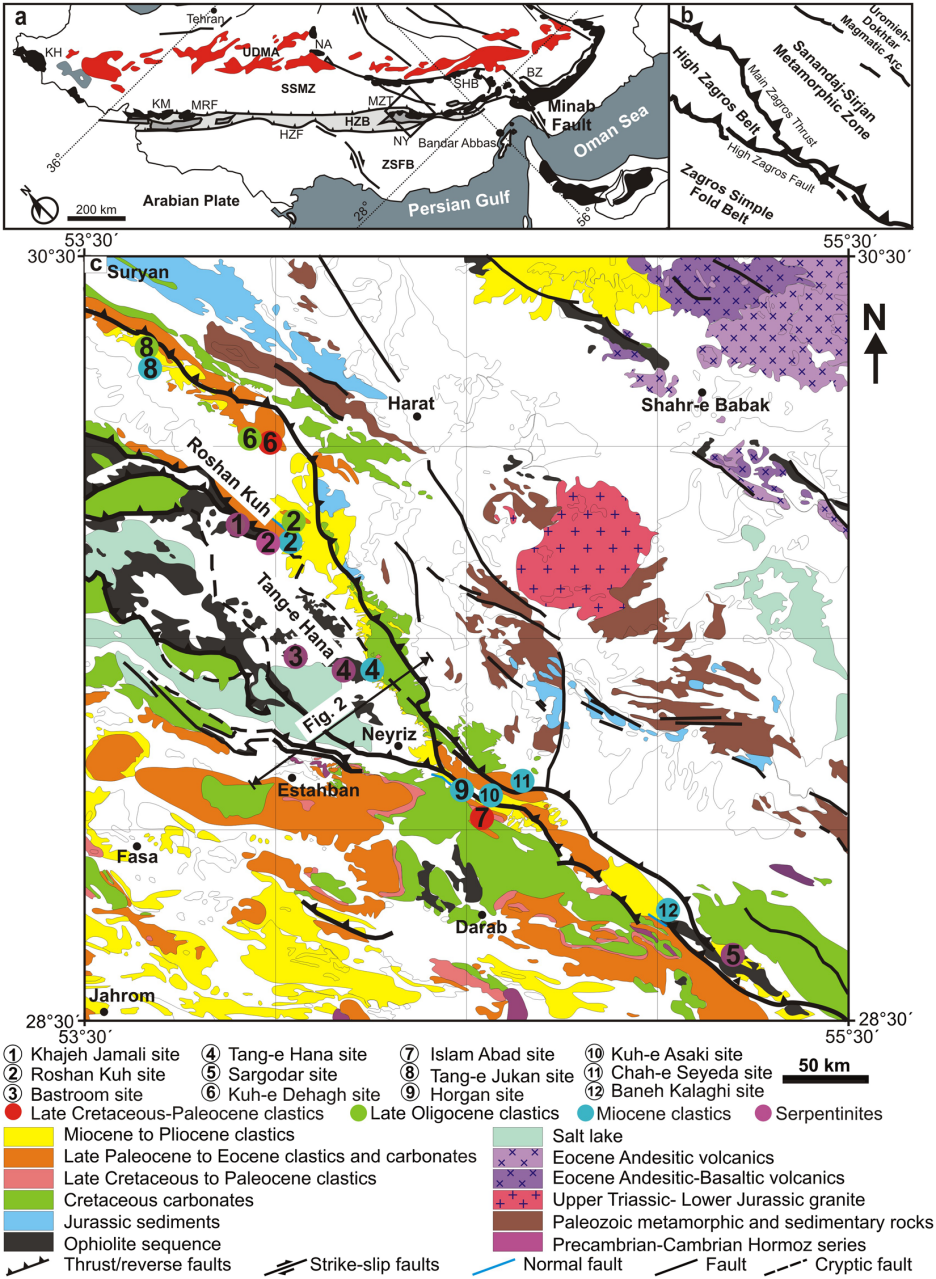
(**a**) Regional map of Iran (modified from refs 16 and 64). Qaterangle: Location of the geological map illustrad in (**b**) and (**c**). KM: Kermanshah ophiolite, NY: Neyriz ophiolite, SHB; Shahr-e Babak ophiolite, KH: Khoy ophiolite, NA: Naien ophiolite, BZ: Bazman ophiolite, UDMA: Uromieh-Dokhtar Magmatic Arc, MZT: Main Zagros Thrust, MRF: Main Recent Fault, HZF: High Zagros Fault, SSMZ: Sanandaj-Sirjan Metamorphic Zone, HZB: High Zagros Belt, ZSFB: Zagros Simply Foldded Belt. This image is modified by Gholami Zadeh, p.; **b**) Simplified map of the geologic zones of the study region was created by Gholami Zadeh, P.; **c**) Simplified geologic map of the sampling sites (modified from ref. 65).The sites have been arranged in 1–12 number with different colors so that the Late Cretaceous-Paleocene clastics are red, Late Oliogcene clastics are green, Miocene clastics are blue and Serpentinites are purple.Location of the Fig. 2 cross section is also given. The normal fault has been shown as a blue small line in the site 2,9 and 12. The map was modified by Gholami Zadeh, P. using CorelDraw x3 v.13 software (http://www.corel.com/content/cgsx3/sp2/readme_en.html).

The Zagros Orogeny, an area with many regular and open anticlines and synclines located in southern Iran, borders the Iraqi Kurdistan toward northwest and the Minab fault toward southeast near Bandar-Abbas. Main Zagros Thrust separates the tectono-stratigraphic zone of Zagros from the Sanandaj-Sirjan metamorphic rocks in the northeast (Fig. 1). The collision between the Arabian and Iranian Plates and the closure of Neotethys Ocean formed Zagros Foreland Basin (e.g. refs 14 and 16). Many researchers have discussed the formation, evaluation and closure of the Neotethys Ocean (e.g. refs 14, 15, 17–20, 22, 23, 25 and 30). The Neotethys Ocean started to form in the Triassic^17, 18^ and began to close with the subduction of the Arabian Plate under the Iranian Plate in the Jurassic due to the opening of South Atlantic^31^. The emplacement of the Neyriz ophiolite on the continental sediments of the Arabian Plate occurred in the Late Cretaceous^32–36^. Based on the hornblende dating in the amphibolites and granulites in the basal thrust sheet of the Neyriz ophiolite, this ophiolite was emplaced in ca. 95 Ma^22, 25, 37^. Based on the intrusion age of the basaltic sheeted dikes (83.6 ± 8.4 Ma: ref. 35), the cooling age of plagiogranites (92.07 ± 1.69–93.19 ± 2.48 Ma: refs 38 and 39), and the age of hornblende in mylonitic amphibolite (94.9 ± 7.6 Ma: ref. 35) in the Tang-eHana ophiolite sequence (Fig. 1c), Babaei *et al*.^17^ have concluded that the Tang-eHana ophiolite was a paleo-mid-ocean ridge which was created, cooled and thrust during a short period of time and/or the basalts were formed in a fore-arc setting and thrust in an accretionary prism in the Late Cretaceous (e.g. refs 17, 40 and 41). Some researchers have posited that the Neyriz ophiolite has been obducted in the Late Cretaceous^32–36^; however, recently it has been reported that the Kermanshah ophiolite formed at 35.7 ± 0.5 Ma based on the zircon dating of the gabbros and plagiogranites in the Kermanshah region^30^ (Fig. 1a). This younger age indicates that the ophiolite could not has obducted before 36 Ma.

The first sedimentary unit deposited after the ophiolite obduction is the Maastrichtian limestones (Tarbur Formation). It has unconformably covered the radiolarites and mafic/ultramafic rocks of the Neyriz ophilites in some parts^17, 18^ (Fig. 1c). Later, as a result of relative sea level fall, the red shales, sandstones, conglomerates and evaporates with Maastrichtian-Paleocene age (Sachun Formation) unconformably deposited on the Cretaceous limestones^18^ which is more terrigenous contents towards NE^16^. The relative sea level rise during the Late Paleocene to the Late Eocene has caused gradual changes to the shallow marine carbonates (Jahrum Formation) which unconformably covered the older rocks including ophiolite segments^16^ (Fig. 1c). The Eocene carbonates are uplifted, folded and outcropped at the end of Eocene^21, 42, 43^. This unconformity in the Late Eocene to Oligocene sequences is attributed to the flexural deformation of the fore-bulge^44^. Later, coarsening upward clastics with Oligocene-Pliocene age deposited on the Eocene carbonates with a sharp boundary at the northeast of Zagros Orogeny^16, 43, 45^ (Fig. 1c). Finally, the Late Pliocene-Quaternary conglomerates cover the older Zagros sediments with an angular unconformity^42, 46^.

There are two major viewpoints about how and when the collision occurred: Alavi^18, 36^ according to the tectono-stratigraphic evaluation of the Zagros Basin interests that the Neyriz and Kermanshah ophiolite obductions occurred in pre-Late Cretaceous, but the collision began in the Late Cretaceous^18, 36^. However, Agard *et al*.^22, 25^, according to the deformation and geodynamic styles, suggest that the collision started in the Late Eocene-Oligocene, and the sedimentary sequences of Zagros Basin folded and uplifted during the Miocene Period^21–27^.

### Detrital Mode through Different Periods

We have studied the mode changes of the sandstone components for understanding the provenances of the Paleocene to the Miocene sandstones by ternary diagrams (QtFL and QmFL: 4) in this section. In addition, we have emphasized on the petrological and mineralogical characterizations of the serpentinite lithic grains and detrital Cr-spinels in order to identify the ophiolite tectonic setting and it changes through consecutive periods. While the serpentinite was in an accretionary prism, the metamorphic deformation affected them. They indicate the foliated and oriented fabrics^47^; therefore, they shed serpentine schist lithic grains when outcropped. However, if the ophiolite obduction occurred, the serpentinite lithic grains display more preserved texture^47^ (cellular and mesh texture).

#### Paleocene sandstones

**Figure 2:**
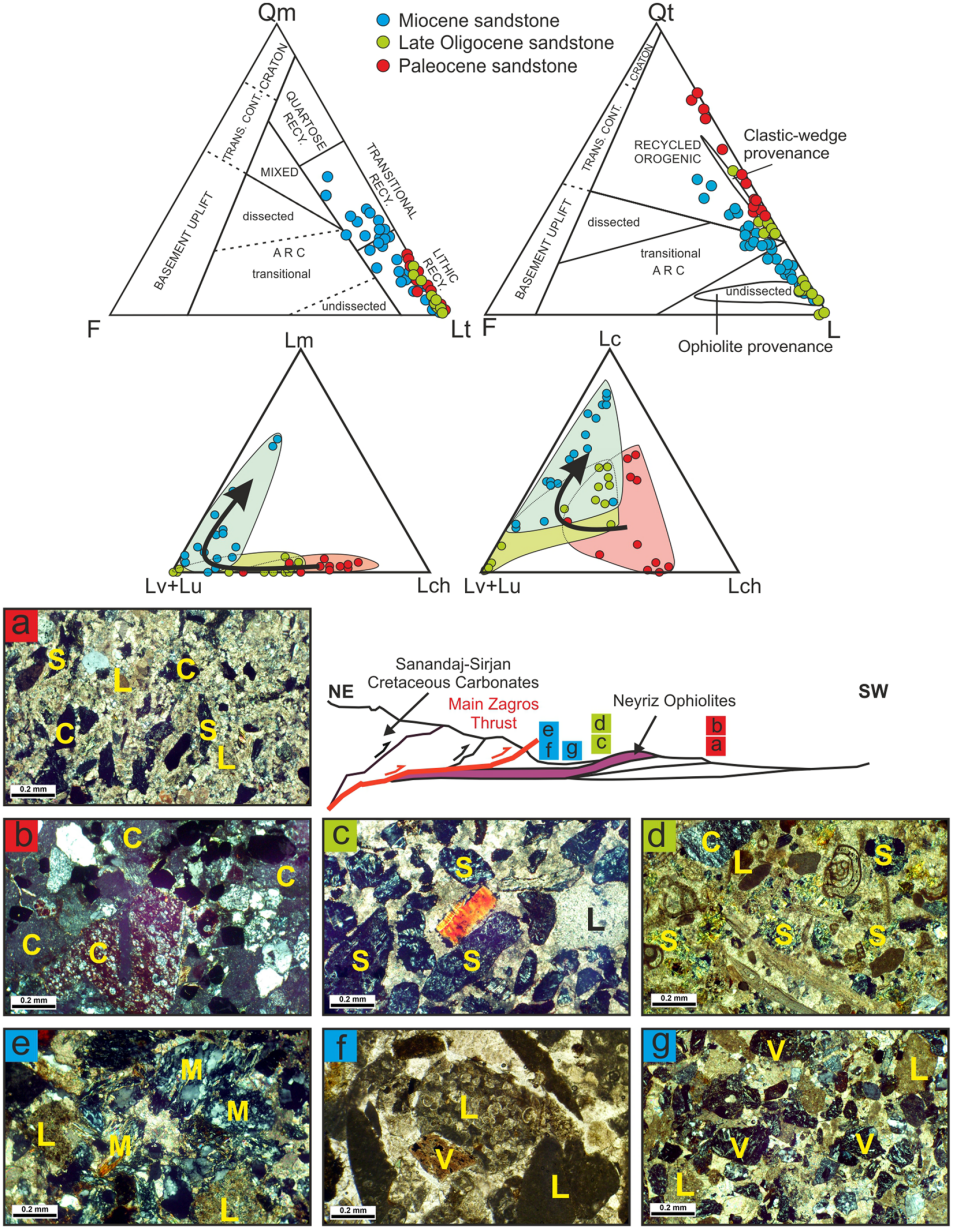
Point counting results on QmFLt and QtFL diagrams^4^, Lc-(Lv + Lu)-Lch and Lm-(Lv + Lu)-Lchternary diagrams (fields on the QtFL based on ref. 2. (**a**,**b**) The Paleocene litharenites containing chert, carbonate and serpentinite lithic grains; (**c**,**d**) The Late Oligocene litharenites containing serpentinites, carbonate and chert lithic grains; (**e**–**g**) The Miocene litharenite and calclithite composed of volcaniclastic, carbonate, slate, siltstone, chert and serpentinite and metamorphic lithic grains. For abbreviations see Table 1. The schematic model indicates the Paleocene sandstones deposited in the SW of ophiolite terrains (Cretaceous Foreland Basin System), but the Late Oligocene and Miocene sandstones are deposited in the NE of ophiolite terrains (Miocene Foreland Basin System). Q = quartz, Qm and Qt were calculated according to Dickinson 1985, F = Feldspar, Lv and V = volcaniclastic rock fragments, Lc and L = carbonate rock fragments, Lch and C = chert rock fragments, Lm and M = metamorphosed sedimentary rock fragments, Lu and S = ultramafic (serpentinites) rock fragments.

**Figure 3:**
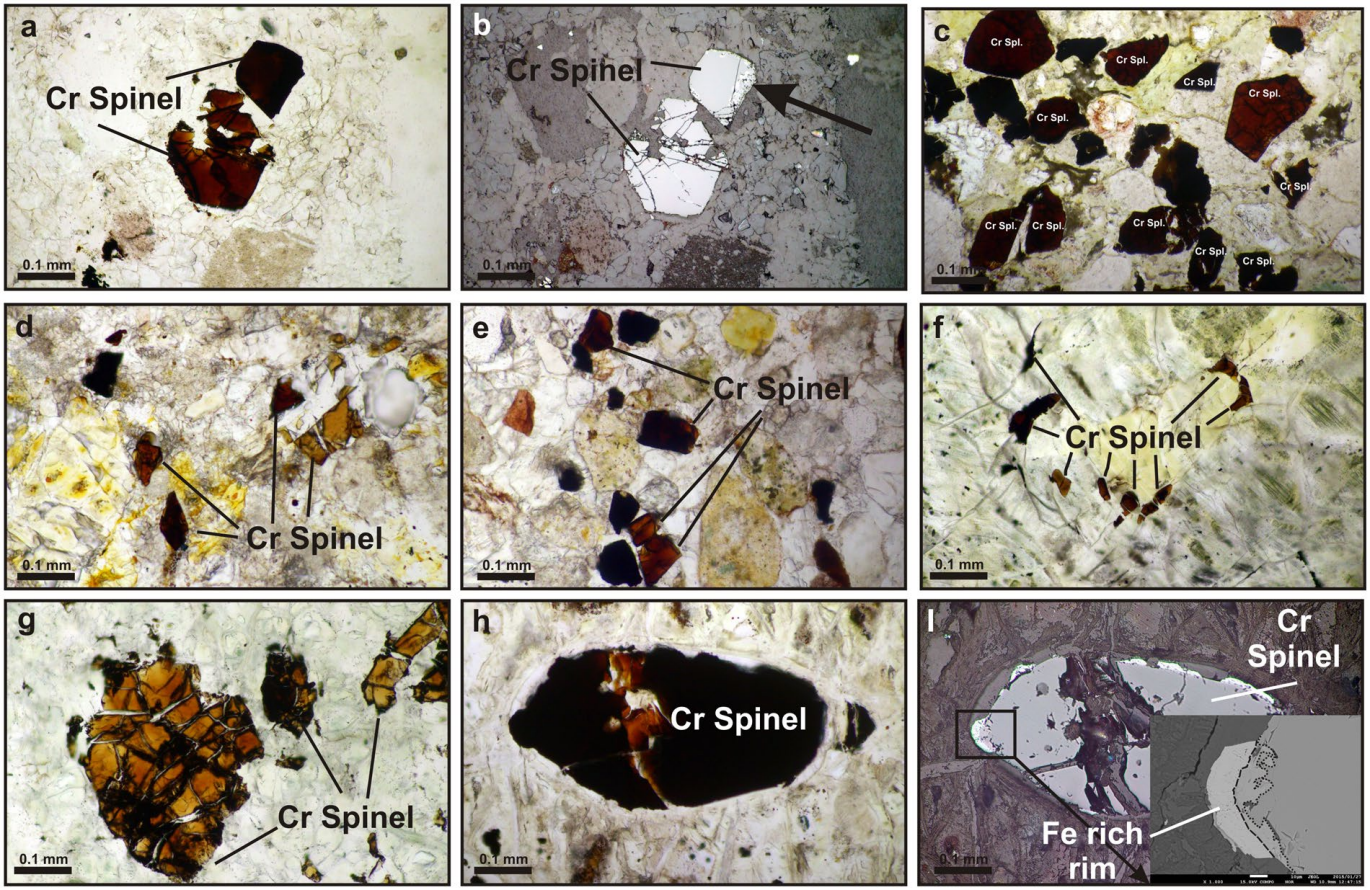
Cr-spinels in different rocks in Neyriz region. (**a**) Detrital Cr-spinels in the Paleocene sandstones with reddish dark brown color; (**b**) Image A in reflected light showing reacion rims; (**c**) High concentration of detrital Cr-spinels in the Paleocene chertarenite at Kuh-e Dehagh with very dark brown to reddish dark brown color; (**d**) Detrital Cr-spinels in the Late Oligocene clalclithite at Tang-e Jokan with broader spectrum of yellow to reddish brown and dark brown color; (**e**) Detrital Cr-spinels in the Miocene litharenite at Roshan Kuh which are similar to Late Oligocene ones; (**f**) Vermiculate Cr-spinels in dunite-harzburgite of Sargodar which have a different color and shape; (**g**) Cr-spinels in the serpentinized dunite-harzburgite at Tang-e Hana which are yellowish brown with oxidized rims; (**h**) Cr-spinels in the serpentinite at Roshan Kuh are reddish dark brown; (**i**) Image **h** in reflected light that the margins of Cr-spinels have been melted. Mg content in the reactive margins increased toward oxidized rims based on the geochemical data.

The point counting samples were chosen from the green medium- to coarse-grained sandstones (Fig. 2a,b). Almost all of the quartz grains are monocrystalline with undulose extinction. The total feldspar content is relatively low, as the feldspar/quartz ratio ranging from 0.01–0.3 and plagioclase is more than orthoclase. The rock fragments are the most abundant grains and generally include (Table 1) chert (Lch), limestone (Lc) and volcanic-ultramafics (Lv-Lu). Most of the ultramafic lithic grains were serpentine-schist lithic grains. The dominant heavy mineral is the detrital Cr-spinels with brownish red to dark red color, similar to what has been observed in the conglomeratic facies (Fig. 3a–c). The detrital Cr-spinels have uniform colors and exhibit analtered rim (Fig. 3b). The sandstones (litharenite, chertarenite) are poor to moderately sorted generally sub-angular grains cemented by carbonates.

**Table 1:** Summary of the point counting results; Q = quartz, F = Feldspar, Lv = volcaniclastic rock fragments, Lc = carbonate rock fragments, Ls = siltstone and mudstone rock fragments, Lch = chert rock fragments, Lm = metamorphosed sedimentary rock fragments, Lu = ultramafic (serpentinites) rock fragments, Qm and Qt were calculated according to Dickinson^4^.

Unit	Age	No.	Q	F	Lv	Lc	Ls	Lch	Lm	Lu	Qm	Qt	TOT
Miocene clastics	Miocene	3	69	48	91	147	7	24	6	3	16.2	23.5	395
Miocene clastics	Miocene	4	35	4	93	88	6	90	1	6	9.91	38.7	323
Miocene clastics	Miocene	7	41	6	133	98	12	5	6	3	10.6	16.5	310
Miocene clastics	Miocene	10	40	18	143	102	6	5	18	2	12	13.5	334
Miocene clastics	Miocene	18	72	17	81	151	12	11	14	—	19	23.2	358
Miocene clastics	Miocene	20	11	4	49	210	30	9	—	1	3.18	6.37	314
Miocene clastics	Miocene	21	41	12	64	178	8	1	1	8	12.5	13.4	313
Miocene clastics	Miocene	30	3	4	46	177	54	12	7	—	0.99	4.95	303
Miocene clastics	Miocene	31	13	4	70	189	11	12	3	3	3.61	8.2	305
Miocene clastics	Miocene	32	7	—	44	233	9	7	—	6	2.29	4.58	306
Miocene clastics	Miocene	34	133	45	38	15	2	7	61	5	40.2	45.8	306
Miocene clastics	Miocene	35	85	30	79	58	5	3	42	3	27	28.7	307
Miocene clastics	Miocene	37	119	31	106	28	2	2	26	1	31.8	34.8	305
Miocene clastics	Miocene	42	107	34	127	35	—	—	6	—	33.7	34.6	309
Miocene clastics	Miocene	45	58	21	32	123	14	6	47	3	16.4	23.4	304
Miocene clastics	Miocene	46	101	11	66	82	—	8	36	—	32.6	38.9	304
Miocene clastics	Miocene	47	19	18	94	119	4	12	26	10	5.96	10.6	302
Miocene clastics	Miocene	58	54	9	115	88	9	11	18	2	15.4	21.6	306
Red beds-RoshanKuh	Late Oligocene	96	—	—	—	4	—	—	—	315	0	0	319
Red beds-RoshanKuh	Late Oligocene	227	—	—	—	7		1	—	304	0	0.32	312
Red beds-RoshanKuh	Late Oligocene	228	18	—	—	2	—	2	—	255	5.81	6.45	310
Red beds-RoshanKuh	Late Oligocene	229	—	—	—	3	—	14	—	286	0	0.99	303
Red beds-Tang Jukan	Late Oligocene	325	28	—	—	131	—	51	—	96	8.82	25.82	306
Red beds-Tang Jukan	Late Oligocene	330	50	—	—	52	—	98	—	101	16.23	48.05	308
Red beds-Tang Jukan	Late Oligocene	331	13	—	—	84	—	44	—	161	4.27	18.75	304
Red beds-Tang Jukan	Late Oligocene	332	23	—	—	115	—	72	—	92	7.61	31.46	302
Red beds-Tang Jukan	Late Oligocene	333	16	—	—	108	—	69	—	109	5.30	28.15	302
Red beds-Tang Jukan	Late Oligocene	334	12	—	—	112	—	81	—	98	2.64	30.69	303
Red beds-Tang Jukan	Late Oligocene	335	7	—	—	95	—	78	—	123	2.31	28.05	303
Red beds-Tang Jukan	Late Oligocene	336	31	—	—	126	15	61	—	83	6.01	29.11	316
Red beds-Tang Jukan	Late Oligocene	371	27	—	—	84	—	87	—	106	8.88	37.5	304
Sachun Fm.	Paleocene	410	28	6	46	140	4	79	—	12	7.94	33.97	315
Sachun Fm.	Paleocene	412	28	11	39	137	6	77	1	8	8.79	34.31	307
Sachun Fm.	Paleocene	413	30	5	76	83	3	83	—	9	9.87	37.17	304
Sachun Fm.	Paleocene	414	49	3	50	102	5	90	—	5	16.12	45.72	304
Sachun Fm.	Paleocene	415	54	3	47	102	5	85	—	11	14.98	45.27	307
Kashkan Fm.	Paleocene	461	36	1	49	—	8	172	—	42	10.06	67.53	308
Kashkan Fm.	Paleocene	463	67	1	36	0	2	159	—	36	20.93	75.08	301
Kashkan Fm.	Paleocene	464	27	9	45	21	6	110	—	88	4.90	44.77	306
Kashkan Fm.	Paleocene	465	9	3	1	66	1	63	—	165	2.92	23.38	308
Kashkan Fm.	Paleocene	466	21	—	—	10	—	186	—	83	4.95	69.31	303
Kashkan Fm.	Paleocene	468	31	2	1	5	—	196	—	70	3.64	75.16	304
Kashkan Fm.	Paleocene	469	17	—	21	35	3	160	—	79	5.45	56.73	315

Petrographic study indicates that these sediments are minerallogically and texturally immature. Comparison between the rock fragment compositions and the outcrop of different terrains on the geological maps (Fig. 1) display their derivation from the ultramafic, radiolarite and limestone rocks. On the Qt_51_F_1_L_48_ and Qm_9_F_1_L_90_ provenance diagrams^4^, almost all samples plot in the recycled orogeny field (Fig. 2). In the collisional belts, both crustal and mantle rocks could be a source for sediments^4^. To recognize and understand the type of orogeny, the lithic grain modes can be plotted in a ternary diagram^2, 3^. If the lithic fragments are plotted on the Lm-(Lv + Lu)-Lch ternary diagram, the samples mostly fall between two Lv + Lu and Lch members (Fig. 2) that may reflect the characterization of the mantel sources or oceanic sediments. In addition, the amount of the chert lithic grains in the Paleocene sedimentary rocks are higher than the other ages (Lc-(Lv + Lu)-Lch diagram in the Fig. 2). The more abundant of serpentine-schist lithic grains relative to the cellular serpentinite lithic grains may reflect the mantel sources have been metamorphosed in a fore arc setting^47^. The study of the components and lithic grains of Amiran Formation with Paleocene age in Kermanshah region indicated the similar results (e.g. ref. 42). Therefore, overall, we concluded that the detrital grains were derived from eroded and recycled sediments of an accretionary prism during the Paleocene. Petrography of the detrital Cr-spinels indicated that they have a uniform color and shape (Fig. 3a–c). In addition, some of them exhibit altered rims that may indicate a partial melting of mantel in the fore-arc setting^48^.

#### Late Oligocene sandstones

The point counting samples were chosen from the red medium to coarse-grained litharenites (Fig. 2c,d). Most of the quartz grains (17% in average) are monocrystalline with undulose extinction. The lithic fragments range from 51% to 99%, which consists of limestones, volcanics-serpentinites, and cherts (Fig. 2 and Table 1). The percentage of chert lithic grains is lower and volcanic-serpentinite grains are higher in Oligocene samples than the Paleocene ones. The cellular serpentinite lithic grains are more relative to the serpentine schist lithic grains. The detrital Cr-spinels with different shapes and sizes are dark red to brownish red, orange and yellow, or even brownish green in the calclithites, and sandy calcarenites and dolomites (Fig. 3d). The sandstones are very poorly to poorly sorted generally sub-angular grains cemented by carbonates and ferruginous materials.

Comparison of petrographic results and the outcrop of different rock terrains on the geological map of Neyriz region (Fig. 1) indicate that the carbonate fragments may have been derived from the Cretaceous and Eocene limestones based on their fossil contents; the volcanic-ultramafic fragments from ophiolite terrains and cherts from radiolarites. According to Qm_22_F_0_Lt_78_ and Qt_5_F_0_L_95_ diagrams^4^, samples all plotted in the recycled orogen field like the Paleocene sandstones (Fig. 2). On the Lm-(Lv + Lu)-Lch and Lc-(Lv + Lu)-Lch ternary diagrams, almost all lithic fragments fall in the sedimentary and volcanic rock fragment parts. In some areas, almost all lithic grains have limestones composition probably derived from the Eocene carbonates, based on their fossil contents. It seems that the lithic grain relative mode changes through space in the Late Oligocene. However, its provenance is similar to the Paleocene ones and probably derived from an oceanic crust, but radiolarite lithic grains had been decreased. The lower amount of serpentine-schist lithic grains relative to cellular serpentine ones may indicate the ultramafic rocks less affected by metamorphism during ophiolite obduction in the Oligocene^47^. The relatively lower amount of K-feldspar, metasedimentary lithic grains and higher amount of cellular serpentinite lithic grains may indicate ophiolite obduction in Oligocene^47^. The color and shape variety of Cr-spinels indicated that they were derived from different ultramafic rocks (Fig. 3d).

#### Miocene sandstones

The samples point counted from the red and green medium to fine-grained sandstones of fluvial and coastal sediments (Fig. 2f,g). The quartz is mostly monocrystalline with undulose extinction and sometimes with corroded rims. The polycrystalline and tectonic quartz grains are rare. The plagioclase with somewhat basic to intermediate compositions and K-feldspars are much more in the Miocene sediments than the other ages (Table 1). The most abundant component is the lithic grains base on their frequency: carbonates, volcanics, and cherts (Fig. 2). The serpentinite lithic grains are relatively low in comparison to the other ages including both serpentine-schist and cellular serpentinite lithic grains. In some areas, the metamorphic sedimentary lithic grains and detrital biotite and muscovite with high percentages were observed. The heavy minerals such as the Cr-spinel, epidote, garnet, amphibole, pyroxene, zircon, tourmaline and glaucophane were also present. The detrital Cr-spinels have a uniform distribution in the Miocene sequence, but with different shapes and colors (brownish red, dark red, brown, orange, yellow) (Fig. 3e). The texture is mostly grain-supported texture characterized by carbonate cement. The roundness of the grains varies from sub-angular to sub-rounded.

On the Qt_22_F_5_L_73_ and Qm_17_F_5_L_78_ provenance diagrams^4^, samples fall in the lithic and transitional recycled orogeny fields like the other ages but with a little difference (Fig. 2). In the Miocene, Qm, F and Lv are more common, so samples shift to Qm on the QmFL diagram and to L on the QtFL diagram. The lithic fragments fall more within the volcanic rock fragment parts and some toward metamorphic corner and far away from the chert rock fragments on the Lm-(Lv + Lu)-Lch and Lc-(Lv + Lu)-Lch ternary diagrams; therefore, the Miocene sediments have been derived from different origins (Fig. 2). The higher percentage of Lv (magmatic provenance = upper plate) relative to Lu (ophiolite provenance = lower plate) and presence of Lm (axial-belt provenance = upper plate) in Miocene sediments may reveala crustal source in addition to the oceanic provenance in this age^2, 3, 47^ (Fig. 2). Therefore, these sediments record post-collision stage. In addition, different heavy minerals and the color and shape varieties of Cr-spinels indicate that they might have been derived from different ultramafic rocks (Fig. 3e).

#### Peridotites

The ultramafic rocks were studied for Cr-spinelsconsist of dunite, harzburgite and serpentinite. Cr-spinels have different colors and shapes (Fig. 3-f–i). Cr-spinels in harzburgites of Tang-eHana are yellowish brown to orange color with anhedral shape and oxidized rims (Fig. 3g). They are subhedral with reddish brown and altered rims in serpentinites of Roshan Kuh (Fig. 3h,i). In serpentinites of Sargodar, they include dark brown vermiculate crystals beside of subhedral shape (Fig. 3f). The color and shape variety of the Cr-spinels reflect that they have been formed in different conditions^6, 7, 9, 11^.

An issue about the Neyriz ophiolite is whether it was formed in MORB (abyssal) or fore-arc (supra-subduction) tectonic setting. Stratigraphically, obduction (MORB) peridotite is overthrust on the margin of passive plate (lower plate) in the suture zones, but fore-arc (supra-subduction) peridotites are associated with large amount of volcaniclastics of active plate (upper plate)^49^. Blocks originated from the oceanic lithosphere in the obduction settings have a MORB or OIB composition, which could not be associated with upper plate stratigraphy^49^. In addition, MORB peridotites do not exhibit any evidence for being in a fore-arc setting or high P-T metamorphic rocks^49^. The volcaniclastics have been thrust on the ophiolites at Garndane-ye Hasan Abad and Sargodar areas in Neyriz region; Therefore, it could be concluded that the Neyriz ophiolite have been formed in a fore-arc setting.

Previous studies have also indicated that the gabbroic and basaltic rocks from the ophiolite sequence have the island-arc tholeiitic and N-MORB compositions^39^, while the volcaniclastics belonging to the island arc are calc-alkaline at the north of Neyriz ophiolite and Gardaneh-ye Hasan Abad^17, 38, 50^. In addition, major and trace element and Nd isotopic compositions of Neyriz magmatic rocks generally indicated that they are similar to fore-arc basalts associated with the supra-subduction setting^39, 51^.

### Cr- Spinel Geochemistry

There are two models for the emplacement of ophiolite on the continental blocks. In the MORB model (abyssal peridotite), an intra-oceanic thrusting formed near the mid-ocean ridge before obduction of the proto-ophiolite, then ophiolite emplaces onto the craton^6, 7, 12, 52, 53^. In this case, the spinel Cr# value is less than 0.6 preserved their chemical variation expected from the mantel under the mid-ocean ridge sequence^52, 53^. In the fore-arc setting or supra-subduction model, the oceanic crust is formed on the subduction zone^52–55^. The shear deformation at the base of subduction complex releases the heat fluids. The thermal metamorphism and partial melting of mantel under the mid-ocean ridge caused the formation of Cr-spinel with Cr# > ~0.6^53^. Therefore, it could be determined distribution of these two genetic groups through time by variation of the geochemical characterization of Cr-spinels.

For this reason, a total of 695 Cr-spinel grains from litharenites, calclithites and peridotite samples with the age of Cretaceous, Paleocene, the Late Oligocene and Miocene were analyzed using EPMA for geochemical studies of the Cr-spinels. Some analyses are summarized in the Table 2.

**Table 2:** Summary of the Cr-spinels geochemistry in terms of cation. Cr# = Cr/(Cr + Al) atomic ratio, Mg# = Mg/(Mg + Fe^2+^) atomic ratio, Fe^3^# = Fe^3+^/(Al + Cr + Fe^3+^), Al^3^# = Al/(Al + Cr + Fe^3+^). SR: Sedimentary rocks.

Age	Paleocene SR		Late Oligocene SR		Miocene SR		Peridotites	
Grain No.	N463	N412	N332	N371	N199	N232	N89	N392
SiO_2_	0.0048	0.0003	0.0013	0.0014	0.0018	0.0077	0.0007	0.0013
Al_2_O_3_	0.3074	0.4419	0.9743	0.9776	0.8426	0.7367	0.7736	0.8183
TiO_2_	0.0059	0.0024	0.0036	0.0000	0.0006	0.0065	0.0039	0.0004
Cr_2_O_3_	1.4187	1.4793	1.0073	0.9538	1.0877	1.1090	1.0964	1.1085
FeO	1.1903	0.7119	0.3568	0.4552	0.5032	0.7905	0.6549	0.5375
NiO	0.0019	0.0013	0.0023	0.0037	0.0031	0.0007	0.0026	0.0024
MnO	0.0409	0.0120	0.0066	0.0068	0.0080	0.0120	0.0082	0.0092
MgO	0.1529	0.3874	0.6479	0.6177	0.5828	0.3895	0.5192	0.5567
CaO	0.0000	0.0000	0.0035	0.0166	0.0020	0.0098	0.0000	0.0000
Na_2_O	0.0043	0.0002	0.0010	0.0004	0.0012	0.0000	0.0016	0.0010
K_2_O	0.0026	0.0004	0.0003	0.0000	0.0000	0.0008	0.0000	0.0000
Total	3.1297	3.0371	3.0049	3.0332	3.0330	3.0632	3.0611	3.0353
Mg#	0.1480	0.3839	0.6510	0.6154	0.5783	0.3863	0.5116	0.5513
Fe3#	0.9388	0.1531	0.0045	0.0379	0.0422	0.0847	0.0787	0.0459
Al3#	0.0109	0.1948	0.4895	0.4870	0.4181	0.3653	0.3811	0.4052
Cr3#	0.0503	0.6521	0.5061	0.4751	0.5397	0.5500	0.5402	0.5489

The Cr# values in Cr-spinels show depletion degree of magma and high Cr# values indicate increasing degrees of partial melting of mantle^5–8, 11^. The geochemistry of Cr-spinels is important because their compositions are not affected by serpentinization process, except for formation of magnetite or ferrichromite rims. The Cr# value could be varied from 0.10 to 0.59 in abyssal peridotites, while supra-subduction peridotites are the Cr-spinels with Cr# = 0.35 to 0.84^12, 52–54^. These groups overlap at Cr#0.40–0.60, but those samples outside this range can be regarded as diagnostic composition^55^. Ti values in the Cr-spinels are also good indicator for differentiating of mantle peridotites, because the Ti values in olivines near the Cr-spinels are low, so Ti values do not change due to reaction surfaces of crystals^56^. The TiO_2_ value is often lower than 1 wt% in the MORB Cr-spinels^53^, while it could be 1–2 wt% in the fore-arc Cr-spinels^53^. The Al, Mg and Fe values in the Cr-spinels show alteration degree of the peridotites (e.g., refs 11 and 57). The Cr-spinels enriched in Fe and depleted in Al and Mg indicate increasing alteration and metamorphism degrees^11^. Moreover, the Mg values in the Cr-spinels of basaltic and andesitic magmatic arcs are more than the peridotites^5^.

**Figure 4:**
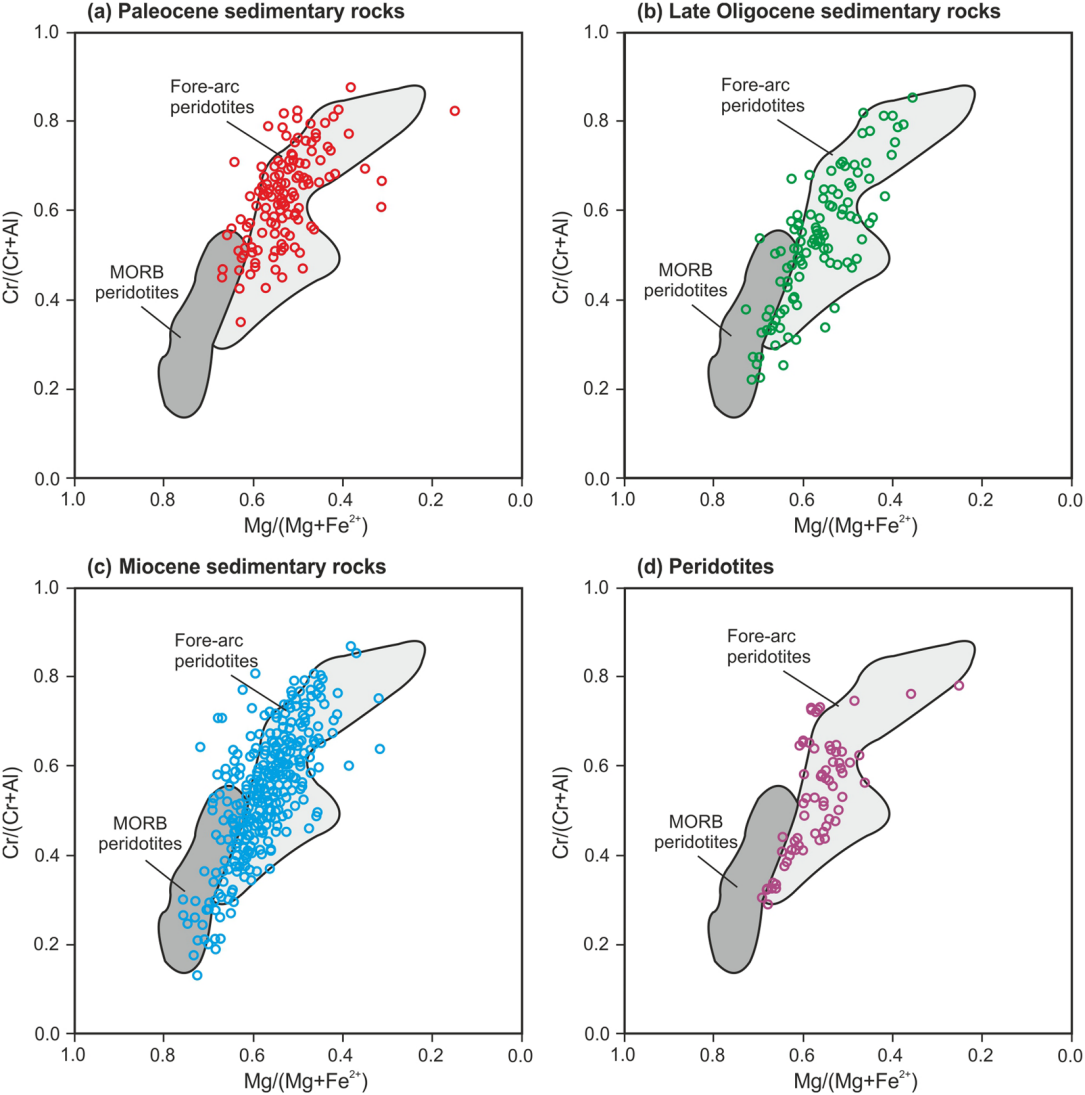
Cr# versus Mg# diagram (fields for Cr-spinels in abyssal peridotites was modified from ref. 12 and for fore-arc peridotites was taken from ref. 52) for the detrital Cr-spinels from Paleocene, Late Oligocene and Miocene sandstones and Cr-spinels of peridotites in Neyriz region. These figures indicate that the detrital Cr-spinels are originated from for-arc peridotites and Cr-spinels of Neyriz peridotites display fore-arc setting. The Paleocene detrital Cr-spinels (**a**) show higher value of Cr# which were derived from more metamorphosed peridotites.

Based on the Cr# versus Mg# diagram (Fig. 4), the Cr-spinels are divided into two groups: The Cr-spinels originated from the abyssal peridotites with Cr# < 0.6 values and the Cr-spinels derived from the fore-arc peridotites with Cr# > 0.6 values^5, 23^. The Fig. 4a indicated that most of the detrital Cr-spinels are derived from the fore-arc peridotites during the Paleocene. The similarity of the Cr# and Mg# values in the Fig. 4b,c indicate that the detrital Cr-spinel originates from the Late Oligocene onwards are similar, thus they may reflect fore-arc origin with lower Cr# values^6, 7, 9^. The Cr-spinels geochemistry of the Neyriz peridotites also indicates the fore-arc composition (Fig. 4d).

Study of major elements of the Cr-spinels from the sandstones and peridotites in the Neyriz region demonstrate that the Cr# value has a better potential to distinguish provenances (Fig. 4)^7, 16^, therefore, the Cr# variations have been used for detection and understanding of the tectonic history of provenances through time as follows:

#### Paleocene sandstones

**Figure 5:**
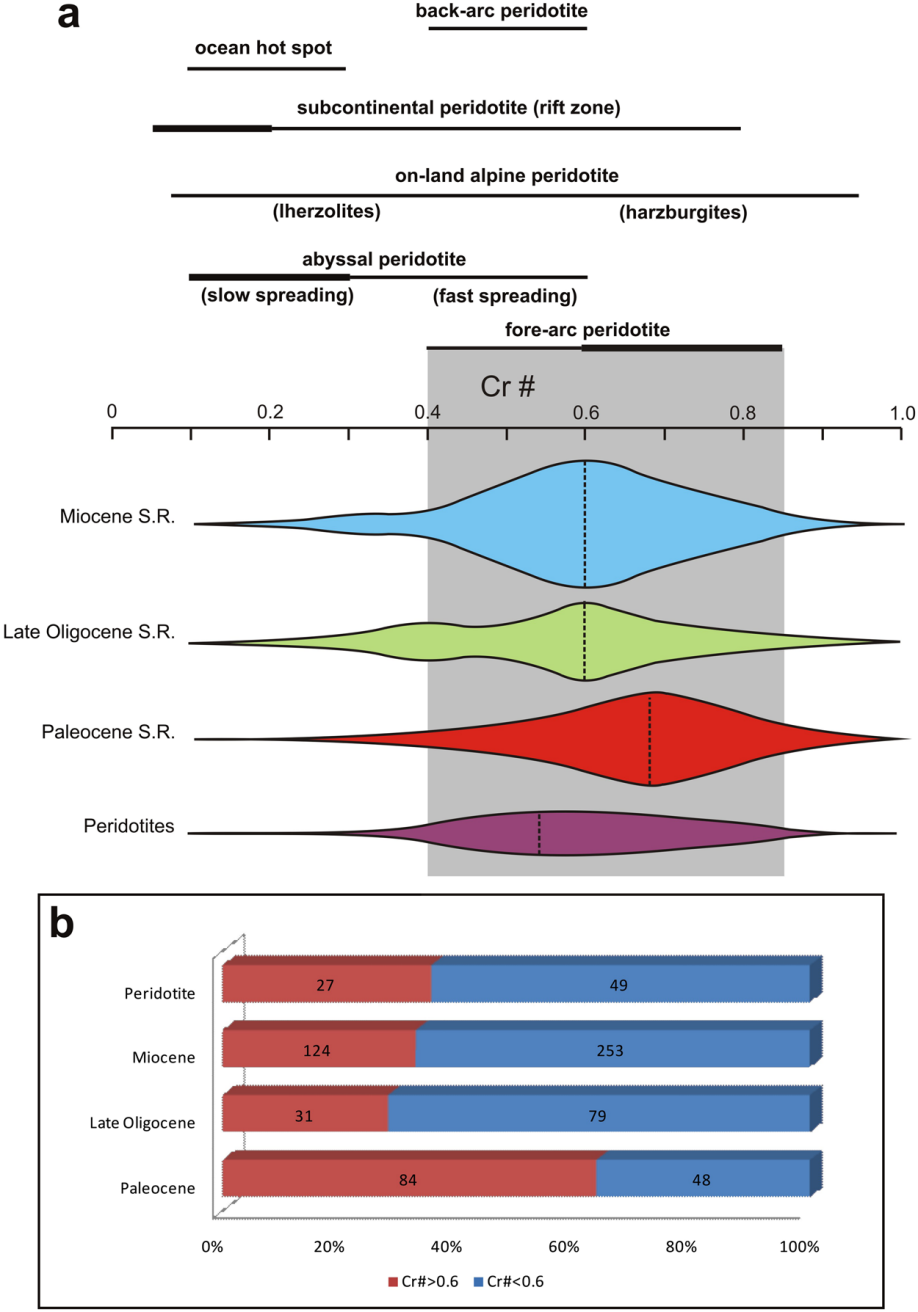
Cr# frequency in detrital Cr-spinels of Paleocene, Late Oligocene and Miocene sandstones and Cr-spinels of peridotites in Neyriz region. Cr# values of peridotites diagram at the top show different tectonic settings from Lee^7^. If we assume that the detrital Cr-spinels originated from peridotites, while according to part **a**, Cr-spinels with Cr# > 0.6 were derived from fore-arc peridotites. The dotted lines on the diagrams show mode. Numbers in the latest diagram indicate the total grains were analyzed. Cr# in the Paleocene detrital Cr-spinels differs from younger ages. These figures indicate that the detrital Cr-spinels were originated from fore-arc peridotite, while Paleocene ones display higher Cr# values relative to the other ages.

A total of 132 detrital Cr-spinels from the Paleocene-Lower Eocene sandstones have been analyzed. Sixty-three of these grains have Cr# > 0.6 (Fig. 5). The Cr# minimum, maximum, and average for detrital Cr-spinels of these sandstones are 0.35, 0.87, and 0.63 respectively. As shown in the Fig. 4 and Fig. 5a,b, most of the Cr# value is more than 0.6. Petrographic studies of Cr-spinels show that they have uniform color (Fig. 3a–c); therefore, detrital Cr-spinels in Paleocene with this chemical composition (higher Cr# values) can be derived from the fore-arc peridotites^6–9, 12^.

#### Late Oligocene sandstones

A total of 110 detrital Cr-spinels from the Upper Oligocene sandstones have been analyzed. Thirty-one of these grains have Cr# > 0.6 (Fig. 5). The Cr# minimum, maximum, and average for detrital Cr-spinels of these sandstones are 0.22, 0.85, and 0.52 respectively. Figure 5a shows two modes and two origins, one at the Cr#~ 0.6 and another one around Cr#~ 0.4 which are generally lower than the Paleocene detrital Cr-spinels. Figure 5b displays a clear change from the Paleocene to the Late Oligocene Cr# of the detrital Cr-spinels. The amount of Cr# < 0.6 (MORB peridotite origin:^6, 7, 9, 53^) is more than the group with Cr# > 0.6 (fore-arc peridotite origin:^6, 7, 9, 53^). The yellowish brown and orange detrital Cr-spinels indicate the lower Cr# values and higher Al# and Mg# values relative to the darker ones. In this study, the detrital Cr-spinels geochemistry of the Late Oligocene indicated they were originated from a MORB to fore-arc peridotites^53^ (Figs 4b and 5a). These compositions are similar to the Cr-spinels geochemistry of the Oman ophiolite, which was obducted on the NE of Arabian Plate; show a transition in the tectonic setting from MORB to fore-arc^58^.

#### Miocene sandstones

A total of 377 detrital Cr-spinels from the Miocene sandstones have been analyzed. One hundred twenty-four of these grains have Cr# > 0.6 (Fig. 5). The Cr# minimum, maximum, and average for the detrital Cr-spinels of these sandstones are 0.13, 0.86, and 0.54 respectively (Fig. 5). Similar to the Late Oligocene Cr-spinels, the Miocene Cr# shows two origins (Fig. 5a); therefore, the Miocene Cr-spinels were derived from the MORB and fore-arc peridotites with different partial melting degrees^6, 7, 9, 53^. Figure 5b indicates the Miocene Cr# values are similar to the Late Oligocene Cr# values.

#### Peridotites

A total of 76 Cr-spinels have been analyzed from the ultramafic rocks (serpentinites, dunites and Harzburgites) in the Baneh Kalaghi, Tang-e Hana, Bastroom, Roshank Kuh, and Khajeh Jamali areas. Twenty-seven of these grains have Cr# > 0.6 (Fig. 5). The Cr# minimum, maximum, and average for detrital Cr-spinels of these rocks are 0.29, 0.78, and 0.53 respectively. The Cr-spinel geochemistry of the Neyriz ophiolite shows a transition from MORB to fore-arc composition such as Oman ophiolite^58^. Comparison between the Cr-spinels of Neyriz peridotites and the detrital Cr-spinels of other ages indicate that the origin of the detrital Cr-spinel from the Late Oligocene onwards is similar to the Cr-spinels of Neyriz peridotites (Fig. 5a,b).

## Discussion

Some authors who have previously researched this topic have concluded that the collision has occurred in the Cretaceous^15, 16, 18, 36^. However some other researchers suggested that it was in the Miocene^30, 59^, and or in the Late Eocene to Oligocene^22, 24–27^. Their reasoning was based on different rheological behavior, plate kinematic evidence, and the forces to drive plate motion along this collisional plate boundary^26^. Alavi^18^ suggests that the ophiolite obduction occurred during 15 My from the Late Turonian to the Maastrichtian; because about 2 meters radiolaritic and ophiolitic polymictic conglomerates have been deposited at the base of the Tarbur Formation (Maastrichtian), and/or the Tarbur Formation covered the Amiran Flysch deposits with marine facies progressively towards the NE. Progradation of the sedimentary facies as a result of thrust loading on the Afro-Arabian passive continental margins is the initial collision (Middle Maastrichtian ~68 Ma)^18^. According to Alavi^18^, the Late Cretaceous- Eocene formations have been formed after collision^36^ and the fore-bulge and back bulge facies are not clear due to lack of the high resolution sedimentology and stratigraphic data from the Late Cretaceous to Middle Eocene.

There are some theories about the regional unconformity from the Upper Eocene to Middle Oligocene strata in High Zagros^28^. Agard *et al*.^22, 25^ posited that the final collision has occurred at this time. In addition, due to similar facies of Zagros and central Iran from Late Oligocene onwards (the Oligocene conglomerate in Zagros is equivalent to the Lower Red Formation in Central Iran and the Burdigalian-Aquitanian limestone in Zagros is equivalent to the Qom Formation in Central Iran), the collision occurred during this time^22, 25^. Another theory is that the collision occurred in Miocene based on the detrital zircon dating of the Cenozoic clastics^19, 20^. Therefore, it is necessary to clear up the tectonic events with newer data.

**Figure 6:**
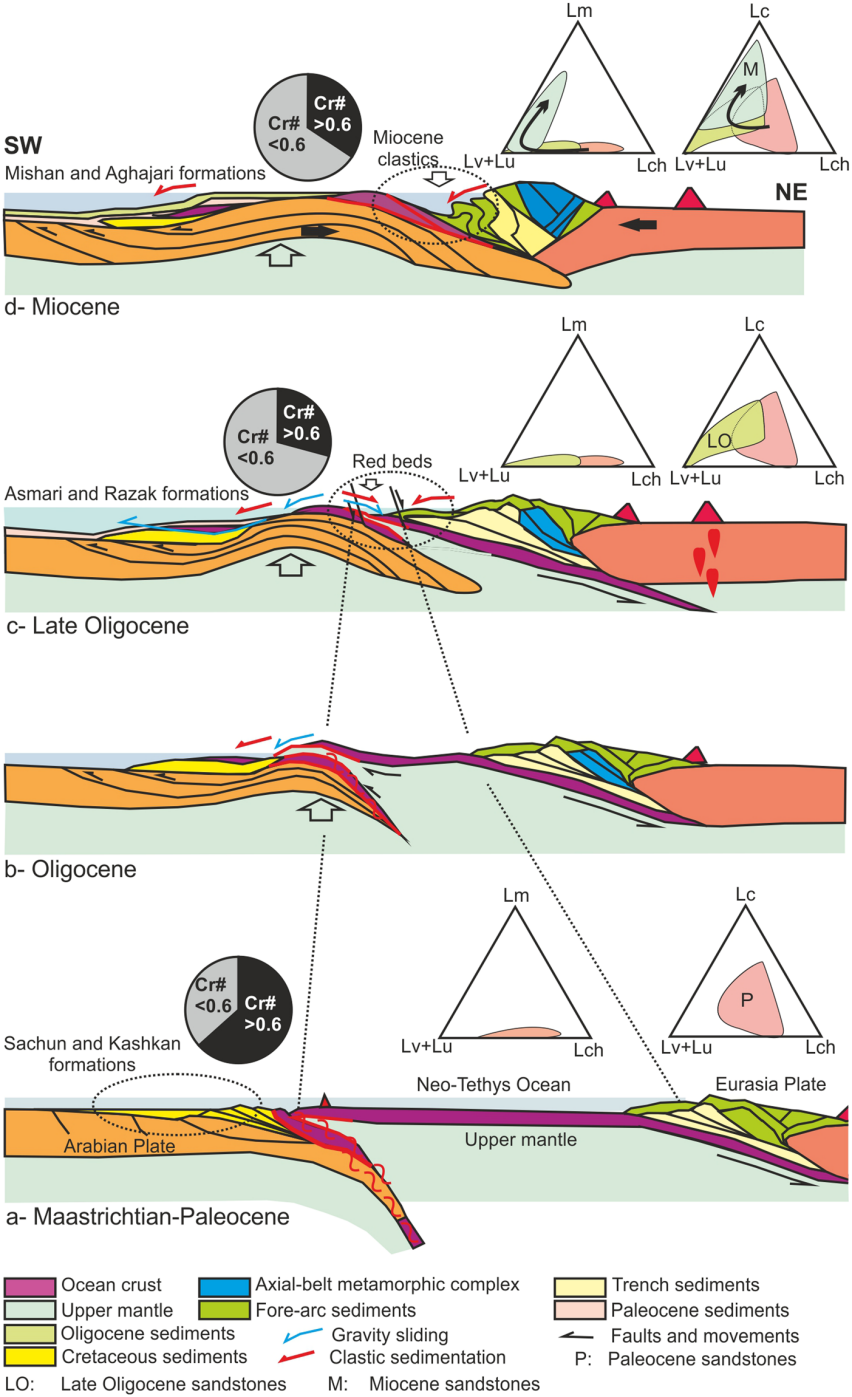
Schematictectonic model for Zagros Orogeny based on Cr-spinel and petrographic studies. Dotted ellipses illustrate the location of samples. See text for more details.

As discussed in the introduction, the study of the sandstone composition can reveal the orogeny type^2, 3^. As in the Oman-type obduction, the origin of sandstone is mostly the ophiolite succession and oceanic crust^2, 3, 47^, but in the Alpine-type collision, the clastics have originated from the upper plate and the continental crust, which contains volcanic rocks from the magmatic arc and the low-grad metamorphic rocks from the axial belts^2, 3^. In the Neyriz region, both events were happening during time. Based on the field observation, microscopic studies and the Cr-spinel geochemistry, the major tectonic historical events in the Neyriz region occurred by detrital mode as follows (Fig. 6).

### Maastrichtian-Paleocene (Pre-Collision stage)

Petrographic studies display the lithic grains in the Paleocene sandstones in Zagros Suture Zone are mostly cherts, limestones and serpentinites-volcaniclastics (Fig. 6a), thus regarding the Fig. 6a,b, it canbe concluded that the upper mantle (serpentinite) and the upper parts of oceanic crust (cherts, limestones, and volcanicalstics) had been outcropped at the NE margin of Arabian Plate (Fig. 2). Of course, some of the Paleocene-Eocene sediments deposited on the Cretaceous nappes in Sanandaj-Sirjan Zone, which have a clear difference in origin from the other contemporaneous sediments. As they are derived from weathering and erosion of the Jurassic-Cretaceous sandstones and limestones of the Sanandaj-Sirjan Zone. Therefore, it may be concluded that there were two separated basins with different provenances; one had been formed on the northern margin of Arabian Plate and another one on the southern margin of the Iranian Plate. The detrital Cr-spinel petrography shows that they are almost uniform color relative to the detrital Cr-spinel in the other ages and they are brown to red in color. The geochemical analysis of the detrital Cr-spinels indicated that they were derived from the fore-arc peridotites^6–8, 58^ and their Cr# values are clearly higher than the ones of the younger ages. The Cr# of Cr-spinels of peridotite has increased due to depletion in the accretionary prism setting (Figs 4–6), as the peridotites are affected by partial melting and infiltrating of H_2_O-rich fluids in detachment area^58^. These fluids are released because of sole metamorphism of oceanic lithosphere along the shear zones^58^ (Fig. 6a).

It seems that the Arabian and Iranian Plates would not have collided yet at this time, because there are no lithic grains of Iranian Plate in the Paleocene sandstones of Zagros Zone (Fig. 6a).Therefore, they originated from erosion and re-sedimentation of the accretionary prism complex from the NE margin of Arabian Plate. After its formation in the fore-arc setting, the Neyriz ophiolite overthrust toward SW onto the Arabian craton, which caused to increasing the height of northern Arabian margin. This thrusting at the N of the Arabian margin leads to the formation of slump and slide structures in an accretionary prism and exposes the upper mantle (Fig. 6b, ref. 60). Based on the isotopic dating, the basaltic rocks in Neyriz ophiolite terrains have been formed in a fore-arc setting and have been shortly thrust on the Arabian Plate toward SW^17, 35, 38, 39, 51^. This basin expanded on the Arabian Plate and SW of the ophiolite terrain. In Eocene, marine transgression resulted in the Jahrum limestones on the Sachun and Kashkan formations and ophiolite mélanges in some areas, even this transgression had continued towards Sanandaj-Sirjan Zone^61^.

### Late Oligocene (Early Collision stage)

Petrographic studies of the Late Oligocene sandstones show that the provenance is similar to the Paleocene age, but generally the chert lithic grains are lower (Fig. 2). The relative frequency of lithic grains changes through space (Fig. 2), which revealsthere were local sub-basins between Arabian and Iranian Plates that were formed due to normal faulting because of local extension forced by underthrusting of Arabian beneath the Iranian plate^26^ (Fig. 6b,c) or slab-break off^22, 25^.

The detrital Cr-spinels are varied in color ranging from yellow to orange, red, brownish red to dark brown. The geochemical analysis of the detrital Cr-spinels shows that they were derived from the fore-arc peridotites^6–8, 58^, but with lower Cr# values relative to the other ages (Figs 4–6) which may be due to exposure of some part of upper mantle with less partial melting. On the NE side of ophiolite, the oceanic crust is tectonically eroded and changed in the second accretionary prism setting on the SE side of it, the oceanic crust with less partial melting is gradually exposed on the Arabian Plate (obduction) and Cr-spinels are released from them (Fig. 6b,c). The Cr-spinel composition of the Neyriz ophiolite and detrital Cr-spinels of the Late Oligocene sandstones are more similar to the Cr-spinel geochemistry of the Oman ophiolite^58^. The Oman ophiolite has been obducted on the NE of Arabian Plate in the Cretaceous (e.g. ref. 47). Therefore, it may be concluded that the ophiolite obduction may have occurred in Oligocene in Neyriz region like Oman because of similar composition of theCr-spinels. In addition, recent dating of the Kermanshah ophiolite illustrated that oceanic crust generation had continued to Eocene^30^, thus the ophiolite obduction occurred after this time. The large unconformity from late Eocene to Late Oligocene may be due to ophiolite obduction and a major change in plate motion in this region. The other point is that the lithic grains are mostly serpentinite and limestone were derived from the oceanic provenance and the lithic grains that originated from the upper plate such as the metamorphic pelitic lithic grains have not appeared yet during the Late Oligocene. Therefore, the Late Oligocene sediments (Fig. 6c) originated from an Oman-type obduction (Oligocene: Fig. 6b) and indicate the early collision stage.

### Miocene (Post Collision stage)

The petrographic studies indicated that during this time, a large amount of the volcanic lithic grains (probably Eocene volcanic eruptions) had been added to the previous provenances (Fig. 2). In addition, the slate and phyletic lithic grains (metamorphosed sedimentary cover of the axial-belt complex) have been uplifted and exposed, so the materials derived from Sanandaj-Sirjan Zone are more than the ones derived from the Zagros Zone (Fig. 2). The signature of metapelitic to metafelsitic lithic grains may represent the erosion of deformed remnants of upper plate during continental collision and Alpine- type orogeny^3, 4, 13, 47^ (Fig. 6d). Petrography of the detrital Cr-spinels shows that they have more variety in color relative to the other ages and their compositions are similar to the Late Oligocene ones. The geochemistry of Cr-spinels indicated that some parts of the less depleted fore-arc peridotite^6–8, 58^ were outcropped at this time. Therefore, the collision occurred before the Miocene and after ophiolite obduction in the Oligocene.

## Conclusions

Based on the sedimentology data on the Cenozoic sequence in the Neyriz region, it can be concluded that:

- From the Late Cretaceous to the Paleocene (pre-collision stage), lithic grains are mostly cherts, serpentinites and carbonates. The detrital Cr-spinel compositions indicate that they originated from the fore-arc peridotites with higher Cr# values that indicate the peridotites with partial melting that were more exposed during this time; thus, sediments have deposited from re-sedimentation of an accretionary prism clastics in the front of it. The general dip direction of the Late Cretaceous-Paleocene basin was to the SW in front of the ophiolite succession.
- The lithic grains mostly consist of limestones, cherts, and serpentinites and their modes locally varied in the Late Oligocene. Geochemistry of Cr-spinels indicates that they have different colors and that their compositions clearly differ from the Late Cretaceous-Paleocene ones; as the Late Oligocene Cr-spinels were derived from the fore-arc peridotites with lower Cr# values. Probably, the normal faults have been formed in the collided zone (Initial collision stage) due to rift inheritance; because of underthrusting of the Arabian beneath the Iranian plate and slab break-off in the Eocene to Oligocene^22, 25, 26^. Therefore, a narrow trough basin (an intermountain basin) has been formed between Zagros and the Sanandaj-Sirjan Zones. After a long-term period of subduction in the Cretaceous, the ophiolite obduction occurred in the Oligocene.
- In the Miocene, the carbonate and volcanic lithic grains were more abundant and some metamorphosed lithic grains were added to the sediment, which may have originated from the axial belt metamorphic complex in the upper plate (post collision stage). The composition of detrital Cr-spinels has not been changed during this time relative to the Late Oligocene ones. The dip of the north flank of the Miocene narrow basin becomes steeper through time (because the basin was received more sediments from the NE margin of the basin) and the basin becomes gradually shallower and folded (Miocene-Pliocene).
- Finally, it is concluded that an accretionary prism has been formed in the Cretaceous at the NE margin of Arabian Plate, which is outcropped and eroded in the Late Cretaceous, ophiolite obduction occurred in Oligocene, and from Late Oligocene to the Miocene, the Arabian and Iranian Plates collided.

## Methods

In this study, samples were taken from the Upper Cretaceous, Paleocene, Upper Oligocene and Miocene sandstones, and the peridotites at different locations in Neyriz regions as follows (Fig. 1):

1. Paleocene sandstones: Islam Abad and Kuh-e Dehagh sites.
2. Upper Oligocene sandstones: Tang-e Jukan, Kuh-e Dehagh and Roshan Kuh sites.
3. Miocene sandstones: Roshan Kuh, Kuh-e Asaki, Baneh Kalaghi, Horgan, Chah-e Seyeda, Tang-e Jukan, Islam Abad sites.
4. Peridotites: Tang-e Hana, Roshan Kuh, Khajeh Jamali, Bastroom, Sargodar sites.

A total of 23 samples from the Late Cretaceous-Paleocene sandstonesand micro-conglomerates (Sachun and Kashkan formations), 33 samples from the Late Oligocene calcareous sandstones and sandy limestones, 246 samples from the Miocene sandstones and 25 samples from serpentinites and serpentinized ultramafic rocks were collected. A total of 300 grains per thin section were counted for 12, 13 and 17 samples from the Paleocene, Late Oligocene and Miocene ages respectively with using Gazzi-Dickinson point-counting method^62, 63^. After preparing polished thin sections, the Cr-spinels were analyzed by EPMA (Electron Probe Micro-Analysis) in the Chemical Analysis Center, University of Tsukuba, Japan. Then, polished thin sections were covered incoal by Vacuum Evaporator equipment JEE-420, and then were analyzed by XA8621 Super Microprobe; JEOL, Tokyo, Japan with a 15 kv potential difference, and 1 A current and 5 micron beam diameter. Elements, which were measured by EPMA, include: Cr, Mg, Mn, Ni, Al, Si, K, Ca, Ti, Fe, Na. 132 grains from Paleocene, 110 grains from the Late Oligocene, 377 grains from the Miocene, and 76 grains from ultramafic rocks were analyzed. The cations and weight percentage of the oxides of elements have been used for data processing.
